# Cardiovascular Wellness—The Role of Lifestyle and Health Equity: A Perspective

**DOI:** 10.1002/hsr2.71230

**Published:** 2025-09-14

**Authors:** Muhammad Umar Soomro, Shahtaj Adil Shah, Gaurav Raj Mishra, Shree Rath, Muhammad Rizwan

**Affiliations:** ^1^ Department of Medicine Jinnah Medical and Dental College Karachi Pakistan; ^2^ Institute of Medicine Tribhuvan University Kathmandu Nepal; ^3^ All India Institute of Medical Sciences Bhubaneswar India; ^4^ Sheikh Zayed Medical College Rahim Yar Khan Pakistan

## Abstract

**Background and Aims:**

Cardiovascular disease (CVD) is a major health concern across the globe, its rising incidence is a result of the interplay of various risk factors. Lifestyle and health status plays a key role in influencing disease progression and outcome. In this perspective, we aim to elucidate the success of lifestyle interventions in decreasing the burden of the disease, while also highlighting the importance of health education and equity.

**Methodology:**

We conducted a literature search on PubMed using key terms such as “cardiovascular health,” “prevention,” “lifestyle interventions,” “exercise,” “health disparities,” and “digital health.” Our search strategy focused on identifying recent studies to ensure the inclusion of current evidence, we supplemented our search with manual citation tracking to ensure comprehensive coverage of the topic.

**Results:**

Diet and exercise significantly affect the pathogenesis of the disease. It was observed that the benefits of exercise were evident when practiced in moderation. Smoking and environmental pollutants are well known for their multiorgan hazardous effects; and the introduction of legislative laws in few countries have proven to be beneficial in reducing disease burden. Measures of health equity and education highlight the prevalent disparities in healthcare provision. These inequalities aren't only influenced by social and educational parameters; studies show that gender plays a massive role as well, and the major reason for this is the lack of diverse clinical trials.

**Conclusion:**

Lifestyle interventions were shown to be highly effective in maintaining cardiovascular health. Fiber‐rich low‐fat meals, and weekly exercise of 2.5 to 5 h help maintain cardiovascular health. The widespread implementation of such interventions is still limited by lack of diverse trials, lacking health education and the prevalent disparity of equitable resource distribution. It is the need of the hour that physicians and policymakers collaborate to ensure a healthy heart among all.

**Abbreviations**
CVD: cardiovascular disease(s)
DASH: dietary approach to stop hypertension
PM: particular matter
SSB: sugar‐sweetened beverages
ASB: artificially sweetened beverages
CHD: coronary heart disease(s)

## 1 Introduction

Cardiovascular disease (CVD) is a global burden and the foremost cause of morbidity and mortality across the world. CVDs represented almost 20.5 million deaths in 2021, resulting in one‐third of all global deaths [1]. The prevailing risk factors for CVD include hypertension, hyperlipidemia, diabetes, smoking, family history of CVD, obesity, and sedentary lifestyle.

In contemporary society, obesity and a sedentary lifestyle pose a significant challenge, superimposed with other conditions; therefore, exercise and a healthy nutritional diet have become the most significant and principal lifestyle interventions to prevent CVD before it progresses as a potential concern. Exercises like running, soccer, basketball, and other group activities have been proven to enhance physical and mental well‐being, reducing stress significantly.

Dietary modifications for a healthy heart include vegetables, fruits, high fiber, and low‐fat content. Recently, there has been an increasing interest in the Mediterranean and DASH diets, as they have a beneficial role in preventing CVD [1].

This perspective examines evidence‐based lifestyle interventions for CVD prevention, including exercise and dietary modifications, further evaluating their impact, barriers, and prospects. We also explore health literacy variables among CVD patients, incorporating psychosocial and educational factors that influence healthcare access. Our goal is to inform healthcare professionals and policymakers at local and regional levels, shaping the development of effective strategies to promote healthy lifestyles among communities and to improve public health outcomes.

## 2 Lifestyle Factors and Cardiovascular Health

Multiple lifestyle factors play a role in influencing cardiovascular health, the primary ones being diet and exercise [2, 3].

Herein, we detail key associations between diet and exercise with the risk of CVD.

### 2.1 Diet and Cardiovascular Health

An important factor contributing to the risk of CVD is diet and culinary habits. Healthy eating patterns have consistently been associated with lower CVD risk; a recent study found a 10%–20% lowering of CVD risk by a 25‐percentile rise in diet scores [4].

The Mediterranean Diet and DASH diet are particularly noted for their preventive roles in CVD, emphasizing plant foods, olive oil, fish, and moderate alcohol while limiting red meat and sweets. Nonetheless, excessive intake of these foods can lead to adverse effects. Sperkowska et al. explored the effects of chocolate and wine consumption on cardiovascular health, mainly focusing on atherosclerosis. While these items are known to affect the cardiovascular system detrimentally, the study found that moderate intake, such as around 30–50 g of chocolate and 130/250 mL of wine for women and men, may confer cardiovascular benefits. However, outcomes depend on age, sex, weight, and existing medical conditions. In addition, concurrent use of cardiovascular medications with these products warrants caution due to potential interactions [5].

### 2.2 Exercise and Cardiovascular Health

The study by O'Keefe et al. found substantial research supporting exercise's therapeutic advantages for cardiovascular health and overall well‐being [6].

Despite the widespread belief that more exercise is beneficial overall, excessive endurance activities could harm cardiovascular health. Evidence reveals that ultra‐endurance events such as long marathons and triathlons can cause acute myocardial damage, indicated by elevated troponin, brain natriuretic peptide levels, and increase the risk of sudden cardiac arrest compared to shorter races. Long‐term, veteran endurance athletes often exhibit abnormal cardiac changes, such as myocardial fibrosis and coronary calcification. Studies consistently link chronic excessive exercise with higher risks of atrial fibrillation and potentially diminishing longevity benefits. Current findings suggest that optimal cardiovascular benefits are likely achieved with 2.5 to 5 h per week of moderate or vigorous physical activity, whereas exceeding 10 h per week may diminish these benefits [6].

A 2022 study on sedentary behavior defined sedentary behavior as “low energy expenditure while sitting or lying down.” Sedentary behavior is an independent risk factor for CVD [7]. Their findings indicate that reducing sedentary time, particularly with walking, shows significant cardiovascular benefits across various health statuses. Interventions targeting sedentary behavior could potentially prevent and manage CVD, although further long‐term research is needed for conclusive guidelines.

Regular moderate exercise is sufficient to effectively lower the risk of cardiovascular events and enhance life expectancy. Intense physical activity, while beneficial, might diminish some advantages seen with less extreme efforts. Current evidence does not strongly support reducing exercise among recreational athletes significantly, if it enhances quality of life or optimizes performance, as risks appear modest and uncertain. Nonetheless, the most significant cardiovascular risk reduction occurs with lower, safer, and more moderate exercise levels [6].

## 3 Effective Lifestyle Interventions

A significant portion of cardiovascular disease is preventable through interventions that target certain lifestyles. Making healthy choices is beneficial for both the body and the mind. Maintaining a healthy weight through regular exercise and physical activity is good for the heart. Smoking is one of the major risk factors for cardiovascular diseases, and cessation improves mortality rates associated with cardiovascular conditions.

Ghazihosseini et al. [8] highlighted environmental pollution as a significant factor contributing to non‐communicable diseases, particularly CVDs. Studies indicate that prolonged exposure to fine particulate matter (PM2.5), prevalent in northern China, escalates the risk of ischemic heart disease and stroke. Vulnerable groups like the elderly, cardiac patients, and obese individuals are particularly susceptible to these effects. Therefore, modern approaches to cardiovascular health must go beyond addressing conventional risk factors and include initiatives for environmental enhancement, emphasizing the need for integrated preventive measures [8].

A meta‐analysis conducted by Yang et al. [9] explored the link between lifestyle behaviors and cardiovascular outcomes, particularly focusing on added sugar, sugar‐sweetened beverages (SSB), and artificially sweetened beverages (ASB).

Data from 109,034 Women's Health Initiative participants examined daily intake levels and conducted Cox regression analyses over 17.4 years. They found that increased added sugar intake (%EAS ≥ 15.0%) was associated with increased risks of total CVD and coronary heart disease (CHD).

Jin et al. [10] proposed an innovative pulse wave analysis technique for public health monitoring, which extracts multiple cardiovascular parameters, such as cardiac output, reflection index, and stiffness index, from the radial artery pulse waveform. The new pulse wave analysis method tested 4374 virtual healthy subjects aged 25 to 75 using pulse waveforms with corresponding cardiovascular parameters. The accuracy rate was 96.347%, with recall, precision, and comprehensive evaluation index F1‐score all exceeding 96%.

Prabhakaran et al. [11] highlighted the role of the DISHA project in imparting lifestyle interventions at individual, family, and community levels. This is achieved through individual counseling, household visits by healthcare workers, the display of posters, and community‐based activities to create and promote awareness. These interventions focused on educating about healthy diets, promoting physical activity, and encouraging smoking and alcohol cessation. Similarly, policymakers in India launched an SMS‐based initiative to promote smoking cessation and diabetes care in their community. 537 participants were randomized between the intervention and control groups, with text messages sent at a frequency and time tailored to the participants' requirements. Results from the trial revealed a reduced incidence of type 2 diabetes in the intervention group (18%) compared to the control group (27%). Follow‐up results years later suggested additional benefits of the intervention in preventing diabetes [11].

## 4 Health Equity in Cardiovascular Care

Addressing health equity in cardiovascular care requires a focused approach to the social determinants of health (SDOH), influencing access to care and health outcomes. Primary care providers (PCPs) are critical in addressing these barriers, facilitating access to specialty care, and ensuring timely diagnosis and management to prevent disease progression. Recent healthcare models, including telemedicine and mobile health clinics, are important tools in reducing these barriers. In particular, patient‐centered homes—where PCPs work closely with specialists through shared electronic medical records (EMR)—enhance communication and create a more holistic approach to patient care. (Brandt el al. 2023) However, cardiovascular health outcomes can vary significantly at the community level. For example, a study by Gerber et al. [12] found that communities with above‐median income had lower mortality rates in patients with acute myocardial infarction (AMI), highlighting the disparity in access to specialized care. Communities with higher income levels tend to have better access to cardiac catheterization and advanced therapies, which are critical in managing acute coronary syndromes. On the other hand, racial and ethnic minorities often face challenges such as lower health literacy and limited access to care, contributing to poorer outcomes, particularly in managing conditions like hypertension [13, 14].

Telemedicine has emerged as a promising solution to overcome these barriers. By enabling remote monitoring of blood pressure and allowing for more frequent medication adjustments, telemedicine helps to mitigate the need for in‐person visits. This not only reduces transportation costs and time but also ensures more consistent management of hypertension, improving long‐term cardiovascular health [15]. The National Heart, Lung, and Blood Institute (NHLBI) has been instrumental in funding community‐based studies to evaluate cardiovascular disease (CVD) risk reduction strategies, particularly for underserved populations [16]. Notable projects like the Stanford Five‐City Project and Minnesota Heart Health Program have demonstrated effective methods for CVD prevention. The NHLBI has also developed culturally tailored educational materials, such as the “Every Heartbeat is Life” program, aimed at improving heart health education in African American communities. Community health workers (CHWs), including Promotores de Salud in Hispanic communities, are particularly effective in bridging the gap between healthcare systems and underserved populations. The HEART trial, for instance, demonstrated that using CHWs can reduce CVD risks in at‐risk Latino populations. In addition to these community efforts, national public health initiatives like Million Hearts and the Community Transformation Grants focus on improving heart health through community‐driven prevention. Further expanding healthcare access in underserved communities is essential, and home blood pressure monitoring (HBPM) has proven to be an effective strategy. Programs like the American Society of Hypertension's (ASH) and Hypertension Community Outreach program, distribute BP monitors to underserved individuals, particularly in Spanish‐speaking populations, while providing multilingual educational materials. This approach empowers patients to monitor their blood pressure at home, leading to better adherence to treatment plans and improved health outcomes [16].

### 4.1 Role of mHealth Technologies in Advancing Cardiovascular Health Equity

Hypertension (HTN) is a leading cause of cardiovascular disease worldwide, but despite effective treatments, many people fail to control their blood pressure. A scoping review conducted by Clifford et al. [17] examines the current state of precision health research using digital tools to manage HTN in adults. The study found that most research focuses on personalized digital interventions (74%), followed by prediction models (17%), and phenotyping (9%). While most studies were conducted in North America and Europe, there were significant gaps in research from Africa and Oceania, regions with high hypertension prevalence [17]. Additionally, more than 60% of studies were from the medical and health sciences, highlighting a need for interdisciplinary collaboration, particularly with fields like engineering and social sciences. Demographic reporting was insufficient, with 45% of studies lacking or partially reporting race and ethnicity data, which raises concerns about the generalizability of findings. Furthermore, the review found the underuse of wearable devices and a lack of integration of diverse data sources, such as social determinants of health and digital literacy, in personalized interventions. The findings emphasize the need for clearer definitions of personalization, more inclusive data, and greater interdisciplinary efforts to advance digital precision health for hypertension management.

The newly developing mHealth (mobile health) technologies can improve the existing inaccessibility of cardiovascular interventions in underserved areas. Mobile applications and smart devices can improve doctor‐patient communication. Digital literacy and commercial disputes could impact the efficacy of these interventions. The literature establishes a need to tailor mHealth technology to improve their cultural relevance and to meet the unique needs of different populations [18]. Mobile health can improve weight management, blood pressure management, medication compliance, and cardiovascular disease risk reduction. Additional research is required to gain better insights on the efficacy of mHealth in increasing cardiovascular health accessibility. Of note, Australia has been a pioneer in developing telehealth systems due to its vast geography and scattered population. To overcome barriers like long‐distance, physician shortage, health disparities, Australia implemented live remote consultations between rural clinicians and cardiologists located in large metropolitan cities, remote monitoring of cardiac patients such as pacemaker checks, post‐op follow‐ups, transmitting ECG reports from rural care centers to specialists for interpretation [19]. Furthermore, the government and healthcare system has supported telehealth through fundings and research, such that Medicare rebates for telehealth consultations, to evaluate the cost‐effectiveness of telehealth, more so patient satisfaction and clinical outcomes [19]. Similarly, in Denmark, which has a much‐advanced healthcare system and patient access, uses telemedicine as a combination of baseline ECGs and use of air transport to transfer patients to the nearest intervention capable centre, subsequently leading to a decrease in mortality for patients living in the remote and distant areas [20]. In conclusion, evidence supports telehealth in several disciplines of cardiovascular care, particularly in remote areas for prompt management. For indigenous people far in remote areas or minorities who donot have access to healthcare, studies suggest reductions in inequalities to be achieved through telehealth in remote areas.

Policy‐level interventions are required to bolster mHealth, including the compensation for healthcare workers on mHealth. Through removal of barriers and improved inclusivity, stakeholders can improve cardiovascular health outcomes, especially for those that are most vulnerable [21].

### 4.2 Telehealth in Rural Communities

Despite having health insurance, a first world country such as Canada, faces the challenge of providing high‐quality specialty care to large rural areas, with only 3% availability of all specialists in those areas. Telehealth is a favorable approach to improve healthcare access in rural communities by delivering specialized care in real‐time across geographical distances. It has a positive impact on patient and caregiver satisfaction. Telemedicine can reduce financial and time‐related barriers related to travel for patients. Audio or video conference for pretreatment consultation educates and stratifies the patients according to their disease risk. Technologies like wireless vital sign monitoring can be used to monitor patient care based on their cancer therapy. A shared care model for posttreatment can determine the necessity and frequency of ongoing monitoring and follow‐up. Incorporating artificial intelligence (AI) can also enhance care delivery through wearable healthcare technology, rhythm analysis, and blood diagnostics analyzers [22].

The tech industry in India has been crescive in tackling health challenges, particularly in remote areas. Startups have developed remote testing and mobile‐testing services for HbA1c, lipid profiles, creatinine, proBNP, ECG, and spirometry. These services are being offered at a fraction of the cost compared to traditional methods, thereby providing access to underserved communities. Healthcare providers are connected via cloud‐based systems to interpret the data and provide prompt feedback. Policymakers and local health departments should help provide such facilities, especially in underserved communities, to maximize the benefits of such technology and reach the masses through community engagements and referrals [11].

## 5 Implementation Challenges and Strategies

Individual motivation, socioeconomic status, access to resources, education, and cultural norms influence lifestyle interventions. The success of interventions can be enhanced by strategies tailored to individual needs and preferences in these areas. Additionally, community support, workplace policies, and public health policies, as well as urban planning and availability of recreational spaces, play essential roles in promoting physical activity and influencing adherence to lifestyle changes. Public health policies and legislative measures can also drive population‐wide changes.

While many studies depict lifestyle interventions in a population cohort, complete integration into clinical practice still needs to be realized. One of the significant barriers is the inclusivity of these studies. Due to being conducted on a cohort with reasonably similar characteristics, generalized conclusions cannot be drawn [23, 24].

These studies are often conducted over a shorter period, leading to a need for more visualization of broader outcomes due to an intervention [9, 10]. With fewer and widely spaced follow‐up time points, studies often need to catch up on the intricacies of the changes, missing confounders influencing the study outcome [9].

Computational models and AI‐based technologies also need a more extensive data set for training, which may not be feasible to provide in a resource‐limited setting [7]. Apart from the study design, barriers also include clinician and patient acceptance. Many clinicians need to be current with the latest findings [24], and many patients may be reluctant to follow a regimen with sufficient data to back it up. Additionally, a subjective betterment or improvement in lab findings may take time to reflect, leading to a lack of compliance, thus negating the effect of such interventions.

Computational models and AI‐based technologies also need a more extensive data set for training, which may not be feasible to provide in a resource‐limited setting [7]. Apart from the study design, barriers also include clinician and patient acceptance. Many clinicians need to be current with the latest findings [24], and many patients may be reluctant to follow a regimen with sufficient data to back it up. Additionally, a subjective betterment or improvement in lab findings may take time to reflect, leading to a lack of compliance, thus negating the effect of such interventions.

Steps should be taken to overcome these barriers and enhance the scalability of the interventions. The medical community can make efforts to promote multicentric and multinational collaborations, allowing a diverse cohort to participate and analyze the role of other factors in enhancing or reducing the beneficial effect of lifestyle interventions. A well‐planned study design, including frequent time points, can also increase the practicability of trials. Such strategies require a close relationship between clinicians, statisticians, and data scientists. Interdisciplinary collaborations are vital in developing an inclusive and well‐designed study trial [24]. Such collaborations can also keep clinicians in a close loop regarding new ideas and advancements in personalized care, allowing seamless integration into daily clinical practice.

With the doctor‐patient relationship shifting from a paternalistic model to an informative and mutualistic one, adequate information be provided to the patient for a well‐informed decision. The success of a greater number of trials and the personal opinion of doctors can promote a positive attitude towards interventions promoting weight loss or increasing exercise duration. While newer and more complex interventions are developing for CVD management, there is a need to develop sustainable models to maximize the full scope and beneficial outcome on a patient's health. Sustainability can be promoted by legislative interventions and adequate surveillance, as done in Italy for environmental pollution control [8].

It has been reported that most studies do not consider social determinants or the economy. It is essential to account for these factors while trying out new interventions to ensure complete social integration.

### 5.1 Addressing Inequities in Cardiovascular Care

Despite multiple developments in the field of cardiovascular care, disparities are still rampant. It is imperative to analyze the various factors which could lead to an inequitable access of healthcare concerning CVDs. One of the major aspects of promoting health equity is improving health literacy amongst patients. In a study by Cabellos‐Gracia et al. [25] different social and demographic parameters were evaluated to unveil the causative factors of this deep‐seated inequities. All cardiovascular pathologies, including valvular heart diseases and arrhythmias were considered in this study. The study revealed various findings, one of which spoke volumes about the income and social class being a significant barrier to health literacy and access to healthcare. People belonging to the lower socioeconomic class and those with no educational background felt less understood by their physicians as compared to their counterpart middle‐class people and those with higher education. Consequently, they were less able to communicate or participate with physicians actively.

Surprisingly, gender can play a very significant role in determining access, treatment plans, and subsequent efficacy of common drugs. In Cabellos‐Gracia et al. [25] study, women were unable to find information pertaining to good health, thus not knowing how to proceed further with such information. The effect of sex on cardiovascular disease, its initiation, progression, treatment and other aspects are summarized by [26] It should be highlighted that apart from the normal physiological factors that determine varied outcomes in men and women, inequity in treatment, bias from healthcare providers and downplaying of early symptoms of CVD account for gross inequity of healthcare provision among women as compared to men. Despite innumerable studies subtly indicating the ineffective response in women, no guidelines exist which prescribe altered regimens for men and women. This suboptimal response has been seen, not only in routine medications like beta blockers, angiotensin receptor‐blockers and angiotensin converting enzyme inhibitors; but also, in drugs used as prevention therapies such as aspirin and statins.

Several strategies are able to address the prevention of CVD. Brown et al. [27] suggested the use of comic books and resources to promote physical activity among participants. The comic books were culturally tailored for health education to improve diet and exercise, such as planning exercises during the day and dietary changes for the upcoming week. As a result, this facilitated the integration of friendship with exercise, as community health workers encouraged participants to attend group exercises like Zumba, yoga, and walking. 90% of the participants in this study received referrals to improve their diet and exercise schedules, as they were overweight or hypertensive. The results suggested that participants adhered to the comic books and implemented strategies such as decreasing salt consumption and taking medications as indicated. Such low‐cost, effective practices encourage and ensure a healthy status for a community [27].

These studies emphasize the demographic, social and education factors which currently plague the healthcare industry of CVDs. It is essential that healthcare providers be alerted of these glaring disparities so that an affordable, accessible, and efficacious treatment regimen can be planned for patients. Physicians should acknowledge their patients' health literacy levels as to better adopt strategies for a greater understanding and approach. An effective strategy includes open‐ended questions and teach‐back methods to ensure patent comprehension. Health systems and local authorities can collaborate together at community levels to promote health literacy by enhancing high school graduation rates, leading to greater literacy rates [28]. No life is more important than the other, and the need to develop personalized medicine is getting stronger with the increasing burden of CVD globally. A multidisciplinary collaboration between physicians, policy‐makers, statisticians, and administration can help achieve equitable distribution of healthcare resources among the masses.

### 5.2 Future Directions

To reduce the global burden and disparities of CVD, it is essential to address three key domains. First, lifestyle interventions must be tailored according to patients' requirements and cultural contexts. These include adapting to healthy dietary habits (such as DASH diet), engaging in community‐based wellness and exercise groups, for example, walking, yoga, and Zumba. Secondly, to achieve health equity, large scale trials must include women and rural communities, eliminating bias in care and, expanding telehealth and community programs to narrow the gap in care access, particularly to women and underserved/rural communities. Lastly, the state and policy‐makers must address the root causes, such as stringent regulations on environmental pollutants and tobacco products, facilities that improve access to healthy foods by providing subsidies, implementing adequate resource distribution via state sponsored local community persons/NGOs and partnerships with educational institutions to create and spread awareness.

A multidimensional approach is crucial to translating this evidence into sustainable, actionable, and equitable impact particularly in rural communities. particularly in rural communities. (Table 1).

**Table 1:** List of included studies, their findings, and recommended interventions.

Reference number	Included study	Significant findings	Recommended interventions
[1]	Global Burden of Cardiovascular Diseases and Risks Collaboration, 1990‐2021. *Journal of the American College of Cardiology*. 2022	Cardiovascular disease (CVD) is a global healthcare burden and it is the leading cause of morbidity and mortality across the world.	Primordial prevention through public health efforts in reducing air pollution and primary prevention through individual efforts focusing on diet, exercise, and smoking cessation are essential for managing the global health burden posed by CVD.
[2]	Relationship between healthy diet and risk of cardiovascular disease among patients on drug therapies for secondary prevention. *Circulation*. 2012	There is an independent and significant association between adherence to a healthy diet and lower risk of recurrent cardiovascular incidents and deaths among CVD patients on drug therapy for secondary prevention.	Prioritizing robust counselling that emphasizes a diet rich in whole foods to further reduce the risk of future cardiovascular events among CVD patients on drug therapy.
[3]	Physical activity and lifetime risk of cardiovascular disease and cancer. *Medicine & Science in Sports & Exercise*. 2017	Higher levels of leisure‐time physical activity are associated with a significantly lower lifetime risk of cardiovascular disease and total cancer.	Promote and encourage regular, even moderate, leisure‐time physical activity throughout adulthood to reduce long‐term cardiovascular disease and cancer risk.
[4]	Association between healthy eating patterns and risk of cardiovascular disease. *JAMA Internal Medicine*. 2020	Consistent adherence to various healthy eating patterns (e.g., Mediterranean, DASH, prudent) is significantly associated with a lower long‐term risk of total cardiovascular disease.	Promote broad adoption of recognized healthy eating patterns as a cornerstone strategy for primary prevention of cardiovascular disease.
[5]	Cardiovascular Effects of Chocolate and Wine—Narrative Review. *Nutrients*. 2021	Moderate consumption of dark chocolate and red wine may offer some cardiovascular benefits due to their polyphenol content, but their overall effect is complex and context‐dependent.	While rich in polyphenols, dark chocolate and red wine should not be recommended as primary cardiovascular interventions; focus on established healthy dietary patterns and moderation, if consumed.
[6]	O'Keefe EL, Torres‐Acosta N, O'Keefe JH, Lavie CJ. Training for Longevity: The Reverse J‐Curve for Exercise. Mo Med. 2020	Exercise exhibits a “reverse J‐curve” relationship with longevity and cardiovascular health, where optimal benefits are seen with moderate‐to‐vigorous activity, but extreme endurance exercise may lead to diminishing returns or potential risks.	Encourage regular moderate‐to‐vigorous physical activity for maximal cardiovascular benefits and longevity, while cautioning against the potential risks of chronic, ultra‐endurance exercise.
[7]	Sedentary Behaviour—A target for the prevention and management of cardiovascular disease. *International Journal of Environmental Research and Public Health*. 2022	Excessive sedentary behavior is an independent risk factor for cardiovascular disease (CVD) and its comorbidities, regardless of physical activity levels	Actively reduce daily sedentary time through regular breaks, standing, and light movement, complementing formal exercise to improve cardiovascular health.
[8]	The Environmental Pollution and Cardiovascular Risk: The role of health surveillance and legislative interventions in cardiovascular Prevention. *High Blood Pressure & Cardiovascular Prevention*. 2023	Environmental pollution, particularly air pollution and heavy metals, significantly increases cardiovascular disease risk, especially in vulnerable groups.	Effective cardiovascular prevention requires health surveillance and strong legislative action to mitigate environmental pollution.
[9]	Added Sugar, Sugar‐Sweetened Beverages, and Artificially Sweetened Beverages and Risk of Cardiovascular Disease: Findings from the Women's Health Initiative and a Network Meta‐Analysis of Prospective Studies. *Nutrients*. 2022	Drinking more added sugars and sugar‐sweetened beverages increases the risk of heart disease, whereas the consumption of drinks sweetened artificially has an inconsistent or neutral association.	Strongly advise limiting intake of added sugars and sugar‐sweetened beverages for cardiovascular health, and consider artificially sweetened beverages as a less harmful alternative to sugary drinks, though not a healthy choice themselves.
[10]	Pulse wave Analysis Method of cardiovascular parameters extraction for health monitoring. International Journal of Environmental Research and Public Health. 2023	Pulse wave analysis provides an effective, noninvasive method for extracting key cardiovascular parameters crucial for health monitoring and early disease detection.	Incorporate noninvasive pulse wave analysis into health monitoring strategies for early identification of cardiovascular risks and personalized prevention.
[11]	Strategic opportunities for leveraging low‐cost, high‐impact technological innovations to promote cardiovascular health in India. Ethnicity & Disease. 2019	Low‐cost, high‐impact technological innovations offer strategic opportunities to significantly improve cardiovascular health promotion in India.	Invest in and widely implement affordable technological solutions (e.g., mobile health, simple diagnostics) to enhance cardiovascular disease prevention and management in resource‐limited settings.
[28]	Assessing and addressing social determinants of cardiovascular health. Journal of the American College of Cardiology. 2023	Systematically assessing and addressing social determinants of health (SDOH) is fundamental to effectively prevent and manage cardiovascular diseases and achieve health equity.	Integrate comprehensive SDOH screening into clinical practice and develop multi‐sectoral interventions to tackle upstream factors influencing cardiovascular health outcomes.
[12]	Neighborhood income and individual education: Effect on survival after myocardial infarction. Mayo Clinic Proceedings. 2008	Both individual education level and neighborhood income significantly influence long‐term survival rates after a myocardial infarction.	Address socioeconomic disparities, including education and neighborhood resources, as crucial factors in post‐myocardial infarction care and overall cardiovascular health outcomes
[13]	Socioeconomic inequalities in quality of care and outcomes among patients with acute coronary syndrome in the modern era of drug eluting stents. Journal of the American Heart Association. 2014	Significant socioeconomic inequalities persist in the quality of care and outcomes for acute coronary syndrome patients, even in the era of advanced treatments like drug‐eluting stents.	Implement targeted interventions to reduce socioeconomic disparities in access to high‐quality care and improve outcomes for acute coronary syndrome patients.
[14]	Income‐Related Inequity in Initiation of Evidence‐Based Therapies Among Patients with Acute Myocardial Infarction. Journal of General Internal Medicine. 2011	Income‐related inequities persist in the initiation of evidence‐based pharmacologic therapies (e.g., ACE‐inhibitors, beta‐blockers, statins) for acute myocardial infarction, particularly among male patients.	Implement strategies to reduce socioeconomic barriers to medication access and adherence, ensuring equitable initiation of guideline‐recommended therapies post‐myocardial infarction for all patients.
[15]	Worldwide trends in hypertension prevalence and progress in treatment and control from 1990 to 2019: a pooled analysis of 1201 population‐representative studies with 104 million participants. The Lancet.	Global hypertension prevalence remained high and stable from 1990‐2019, but despite increases in treatment, control rates were unacceptably low, especially in low‐ and middle‐income countries.	Prioritize scalable, cost‐effective interventions for hypertension screening, treatment, and sustained control, particularly in low‐ and middle‐income regions to reduce the global burden.
[16]	Community‐Based Approaches to prevention and management of hypertension and cardiovascular disease. Journal of Clinical Hypertension. 2012	Community‐based interventions are effective in promoting hypertension awareness, improving blood pressure control, and modifying cardiovascular risk factors at a population level.	Implement accessible, culturally relevant community programs focused on education, screening, and lifestyle modification to prevent and manage hypertension and CVD.
[17]	Trends and Gaps in Digital Precision Hypertension Management: Scoping Review (Preprint). Journal of Medical Internet Research. 2024	Digital precision health in hypertension primarily focuses on personalized interventions but lacks diverse data integration (e.g., social determinants, physical environment) and consistent reporting of demographic data.	Develop digital hypertension tools with more comprehensive data sources beyond clinical data, improve personalization, and ensure equitable design for diverse populations.
[18]	Mobile health in preventive cardiology: current status and future perspective. Current Opinion in Cardiology. 2021	Mobile health (mHealth) tools effectively support cardiovascular disease prevention by improving risk factor management, medication adherence, and lifestyle modification.	Integrate mHealth applications and wearable devices into preventive cardiology strategies to empower patients in self‐management and enhance adherence to treatment and lifestyle goals.
[19]	The Use of Telehealth to Reduce Inequalities in Cardiovascular Outcomes in Australia and New Zealand: A Critical Review. Heart Lung Circ. 2017	Telehealth shows promise in improving cardiovascular outcomes and reducing healthcare inequalities in Australia and New Zealand, particularly for rural and remote populations, by increasing access to care.	Leverage telehealth to bridge geographical gaps in healthcare access and enhance equitable cardiovascular disease prevention and management in underserved regions.
[20]	Pre‐hospital diagnosis and transfer of patients with acute myocardial infarction‐‐a decade long experience from one of Europe's largest STEMI networks. J Electrocardiol. 2013	Pre‐hospital diagnosis of STEMI and direct transfer to a PCI‐capable hospital significantly reduced time to reperfusion, improving outcomes over a decade.	Implement and optimize pre‐hospital STEMI diagnostic protocols and direct transport systems to specialized centers to minimize treatment delays and improve patient prognosis.
[21]	Telehealth Use to Address cardiovascular Disease and Hypertension in the United States: A Systematic Review and Meta‐Analysis, 2011–2021. Telemedicine Reports. 2023	Telehealth effectively improved intermediate cardiovascular disease and hypertension outcomes, including blood pressure and LDL‐cholesterol, especially among older and male populations, but its impact on major adverse cardiac events is unconfirmed.	Integrate telehealth into cardiovascular disease and hypertension management plans, particularly for at‐risk populations, while continuing research into its long‐term impact on major clinical events.
[22]	Pursuing connectivity in Cardio‐Oncology Care—The future of telemedicine and artificial intelligence in providing equity and access to rural communities. Frontiers in Cardiovascular Medicine. 2022	Telemedicine and artificial intelligence (AI) hold significant promise for improving equity and access to specialized cardio‐oncology care in rural communities.	Strategically integrate telemedicine and AI technologies to bridge geographical gaps and enhance specialized cardiovascular care delivery for underserved rural populations.
[23]	Uses of social Determinants of health data to address cardiovascular Disease and health Equity: A scoping review. Journal of the American Heart Association. 2023	Social Determinants of Health (SDOH) data are increasingly used to identify and address cardiovascular disease (CVD) disparities and promote health equity, but standardization and integration remain challenges.	Systematically collect and integrate SDOH data into CVD prevention and management to tailor interventions that effectively address health inequities and improve population health.
[24]	Achieving optimal population cardiovascular health requires an interdisciplinary team and a learning healthcare system: a scientific statement from the American Heart Association. Circulation. 2020	Achieving optimal population cardiovascular health necessitates an interdisciplinary team approach and a learning healthcare system that continuously adapts based on data and outcomes.	Foster collaborative interdisciplinary teams and develop learning healthcare systems to drive continuous improvement in population‐level cardiovascular health outcomes.
[25]	Relationship between Determinants of Health, Equity, and Dimensions of Health Literacy in Patients with Cardiovascular Disease. International Journal of Environmental Research and Public Health. 2020	Health literacy in cardiovascular disease patients is significantly influenced by social determinants of health and has a crucial impact on health equity.	Tailor health education and communication strategies to account for patients' social determinants of health to improve health literacy and reduce cardiovascular health inequities.
[26]	Closing the sex gap in cardiovascular mortality by achieving both horizontal and vertical equity. Atherosclerosis. 2024	Achieving both horizontal (equal access to care) and vertical (tailored care based on need) equity is crucial to close the persistent sex gap in cardiovascular mortality.	Implement healthcare policies and practices that ensure equitable access to CVD prevention and treatment for all sexes, alongside sex‐specific diagnostic and therapeutic approaches.
[27]	Evaluation of Healthy Fit: A community health worker model to address Hispanic health disparities. *Preventing Chronic Disease*. 2018	The “Healthy Fit” community health worker model effectively improved health behaviors and reduced cardiovascular disease risk factors within the Hispanic population.	Utilize culturally competent community health worker models to address health disparities and improve cardiovascular outcomes in specific ethnic populations.

## 6 Conclusion

The healthcare sector globally faces a challenge, with conditions which stand as the primary cause of illness and death worldwide. Cardiovascular diseases are long term conditions influenced by risk factors and lifestyle adjustments that can help reduce these risks. Factors like blood pressure, high cholesterol levels, diabetes, smoking, family history of heart diseases, obesity, lack of activity pose major risks for cardiovascular diseases. Making lifestyle changes such as following a diet and engaging in exercise can lower the chances of developing heart problems. A heart healthy diet involves consuming vegetables, fruits, foods rich in fiber, and low‐fat options. Exercising for 2.5 to 5 h weekly is beneficial for maintaining good cardiovascular health.

Efforts to promote health through lifestyle changes encounter challenges like patient reluctance to adopt new habits, healthcare providers needing to stay updated on the latest research findings constantly and the need for more studies in this area. These research shed light on the social and educational issues currently affecting the healthcare industry concerning diseases. It is crucial for healthcare professionals to recognize these disparities so they can develop cost treatment plans that are accessible and effective for their patients.

Thankfully these obstacles can be tackled through fostering a bond between doctors and patients and encouraging efforts across multiple centers and countries to create and implement fresh insights on lifestyle interventions. Every life holds value underscoring the growing urgency for tailored approaches, in light of the escalating cardiovascular disease challenges worldwide.

## Author Contributions

**Muhammad Umar Soomro:** conceptualization, writing – original draft, writing – review and editing, supervision. **Shahtaj Adil Shah:** writing – original draft, writing – review and editing. **Gaurav Raj Mishra:** writing – original draft, writing – review and editing. **Shree Rath:** writing – original draft, writing – review and editing. **Muhammad Rizwan:** writing – original draft, writing – review and editing.

## Conflicts of Interest

The authors declare no conflicts of interest.

## Transparency Statement

Gaurav Raj Mishra affirms that this manuscript is an honest, accurate, and transparent account of the study being reported; that no important aspects of the study have been omitted; and that any discrepancies from the study as planned (and, if relevant, registered) have been explained.

## Data Availability

Data sharing is not applicable to this article as no new data were created or analyzed in this study. Data sharing not applicable to this article as no datasets were generated or analysed during the current study.
